# Urinary Tract Infection Molecular Mechanisms and Clinical Translation

**DOI:** 10.3390/pathogens5010024

**Published:** 2016-02-24

**Authors:** Gabriela Godaly, Ines Ambite, Manoj Puthia, Aftab Nadeem, James Ho, Karoly Nagy, Yujing Huang, Gustav Rydström, Catharina Svanborg

**Affiliations:** Department of Microbiology, Immunology and Glycobiology (MIG), Institute of Laboratory Medicine, Lund University, 221 00 Lund, Sweden; ines.ambite@med.lu.se (I.A.); manoj.puthia@med.lu.se (M.P.); aftab.nadeem@med.lu.se (A.N.); james.hcs@gmail.com (J.H.); dr.nkaroly@gmail.com (K.N.); yujing.huang@med.lu.se (Y.H.); gustav.rydstrom@med.lu.se (G.R.); catharina.svanborg@med.lu.se (C.S.)

**Keywords:** immunomodulation, genetics, innate immunity, urinary tract infection, susceptibility

## Abstract

Rapid developments in infection biology create new and exciting options for individualized diagnostics and therapy. Such new practices are needed to improve patient survival and reduce morbidity. Molecular determinants of host resistance to infection are being characterized, making it possible to identify susceptible individuals and to predict their risk for future morbidity. Immunotherapy is emerging as a new strategy to treat infections worldwide and controlled boosting of the host immune defense represents an important therapeutic alternative to antibiotics. In proof of concept studies, we have demonstrated that this approach is feasible. The long-term goal is not just to remove the pathogens but to also develop technologies that restore resistance to infection in disease-prone patients and devise personalized therapeutic interventions. Here, we discuss some approaches to reaching these goals, in patients with urinary tract infection (UTI). We describe critical host signaling pathways that define symptoms and pathology and the genetic control of innate immune responses that balance protection against tissue damage. For some of these genes, human relevance has been documented in clinical studies, identifying them as potential targets for immune-modulatory therapies, as a complement to antibiotics.

## 1. Introduction

Key questions in infection biology have been addressed in the urinary tract and mechanistic insights gained in this model have proven relevant to other infections, as well. Urinary tract infections (UTIs) begin when bacteria gain access to the urinary tract and attack the mucosa in the urinary bladder, ureters and/or the renal pelvis. Symptoms are caused by an excessive host response to infection and an inflammatory tissue infiltrate is the main cause of symptoms and disease. Acute cystitis and acute pyelonephritis can therefore be regarded as infection-driven inflammatory disorders of the bladder and kidneys, respectively. In addition, the urinary tract may host large numbers of bacteria without causing symptoms. In patients with asymptomatic bacteriuria (ABU), bacteria create a symbiotic relationship, which allows for long-term persistence and protects the host against infection with more virulent strains. The challenge is to understand what makes bacteria uropathogens or “commensals” ABU strains, why some patients develop acute pyelonephritis while others develop ABU, and why some individuals are prone to UTI, in the first place. The mission is to use the new molecular tools to counter the burden of disease. Studies from many groups are now bringing us closer to the point of translating into molecular language these conceptual advances and provide suggestions for new therapies and diagnostic markers.

Our strategy has been to elucidate molecular mechanisms of UTI sequentially, from the initial contact of bacteria to the mucosa to the immune response and genetic susceptibility profiles. Our main goals have been to

1. Identify key, virulence factors that distinguish pathogens from commensals.
2. Characterize critical host signaling pathways that define symptoms and pathology.
3. Inactivate specific genes that control these pathways and characterize the consequences for protection and pathology.
4. Confirm the relevance of these genetic variants for disease in susceptible or resistant patient groups.
5. Address how ABU strains achieve the state of “commensalism”.
6. Develop immune-modulatory therapies, as a complement to antibiotics.

This brief review is intended as an introduction to these questions, with some relevant references. The individual papers in this Special Issue of Pathogens, present in greater detail the results from leaders in the field and the resulting advances in understanding of the molecular basis of UTI (Table 1).

## 2. Key, Virulence Factors that Distinguish Pathogens from Commensals

The virulence concept in UTI emerged when it was recognized that specific properties distinguish *Escherichia coli* (*E. coli*) strains that cause severe disease from strains that are carried asymptomatically, in the urinary tract [2]. These “virulence factors” were initially defined epidemiologically, by disease association. Increasingly, it was recognized that virulence reflected direct effects of certain strains on host tissues, and mechanisms for tissue attack began to be analyzed in cellular models. The adhesion of virulent *E. coli* strains provided an early example of pathogen crosstalk with infected host tissues, opening up the field of “cellular microbiology”. Subsequently, P fimbriae were identified as key virulence-enhancing ligands, also useful as markers of virulent strains that cause urosepsis [3,4,5,6].

In parallel with the discoveries of bacterial adhesion, methods were developed to identify and classify strains that cause different forms of UTI and to distinguish the disease isolates from commensal strains in the fecal flora. Interestingly, the molecules chosen for classification were often virulence factors in their own right, acting as endotoxins (LPS, O antigens), bacterial survival enhancers (Capsular polysaccharides, K antigens) and organelles for bacterial motility (flagella, F antigens). The toxin hemolysin was also identified as an early marker of uropathogenic strains, compared to isolates from the fecal flora, as well as aerobactin, one of the first iron-binding proteins to be identified in UPEC [7]. Based on surface antigens or multilocus isoenzyme typing, bacterial lineages or clones were characterized and essential aspects of the population dynamics of UPEC were revealed, including the horizontal transfer of genes involved in adhesion and bacterial adaptation to the urinary tract environment [8,9]. The definition of pathogenic clones and their virulence factors was given a molecular context by the pathogenicity island concept [10], which demonstrated that genes encoding virulence factors in uropathogens were clustered and flanked by insertion elements, indicating that they might have moved, “en bloc”, into the chromosome of ancestral *E. coli* strains and become fixed by the selective advantage afforded by these genes in the infected host [11].

Virulence factors are distinct from the bacterial determinants of persistence, as they are engaged in tissue attack and disease. ABU strains colonize the human urinary tract for extended periods of time and create a state of symbiosis with the host. In epidemiological surveys, about 50% of ABU strains are related to the virulent clones but fail to express virulence factors, due to mutations or deletions that attenuate virulence (see Dobrindt *et al.*, this Special Issue [1]). In addition, ABU strains modify the host environment in their favor by suppressing host gene expression. A reduction in RNA Polymerase II dependent gene expression by >60% was detected already 24 h after bacteria entered the human urinary tract. This included genes that regulate the immune status illustrating how “non-virulent” strains bacteria can tame the antibacterial host defense (see Ambite *et al.*, this Special Issue [1]). These findings change our view on asymptomatic carrier strains from passive bystanders to active participants in host-microbial co-evolution.

For comprehensive updates on bacterial virulence factors and UTI, please see Mobley *et al.* [12] and Hultgren *et al.* [13]. See also Hunstad *et al*., Hultgren *et al.*, Klein *et al*., and Pichl *et al.*, and Mobley *et al.*, this Special Issue [1].

## 3. Critical Signaling Pathways that Define Symptoms and Pathology

The strong association between adherence, virulence and disease severity, indicated that there must be a direct molecular link between the bacteria, their epithelial cell receptors and disease. We provided such a molecular link, by showing that infected epithelial cells produce inflammatory mediators that start the mucosal inflammatory response [14,15]. Surrounding cells and recruited inflammatory cells then take the decisions about the quality and quantity of the mucosal response, leading to disease or protection [16]. The epithelial cells are thus important players in the mucosal immune system; acting both as docking sites for the bacteria and as sensors of microbial attack [17].

The quality of the inflammatory response varies with the virulence repertoire of the infecting strain. It is also determined by the expression of specific host cell receptors and by the signaling pathways that they activate [18,19]. For example, the binding of P fimbriae to glycolipid receptors alerts the host to the presence of virulent bacteria and activates an innate immune response in the urinary tract mucosa [19]. Through sequential activation of TLR4 and the TRIF/TRAM adaptors, this signaling pathway engages MAPKs, p38 and CREB leading to the formation of transcription factor complexes defined by IRF3 and the activation of type I IFN responses. In parallel, phosphorylated FOS and JUN form the AP1 transcription factor complex, which activates chemokine and cytokine production.

Fimbriae–receptor interactions illustrate how signaling pathways in the host are controlled by virulence factors and their receptors. A change from P- to Type 1 fimbrial expression shifts the host response to the MyD88 and TIRAP dependent arm of the TLR4 signaling pathway, leading to NFkB dependent transcription and inflammasome activation [20]. For comprehensive updates on the innate immune response to UTI, please see Godaly *et al.* [16] and Ambite *et al.* [17].

## 4. Inactivate Specific Genes that Control These Pathways and Characterize the Consequences for Protection and Pathology

**Figure 1:**
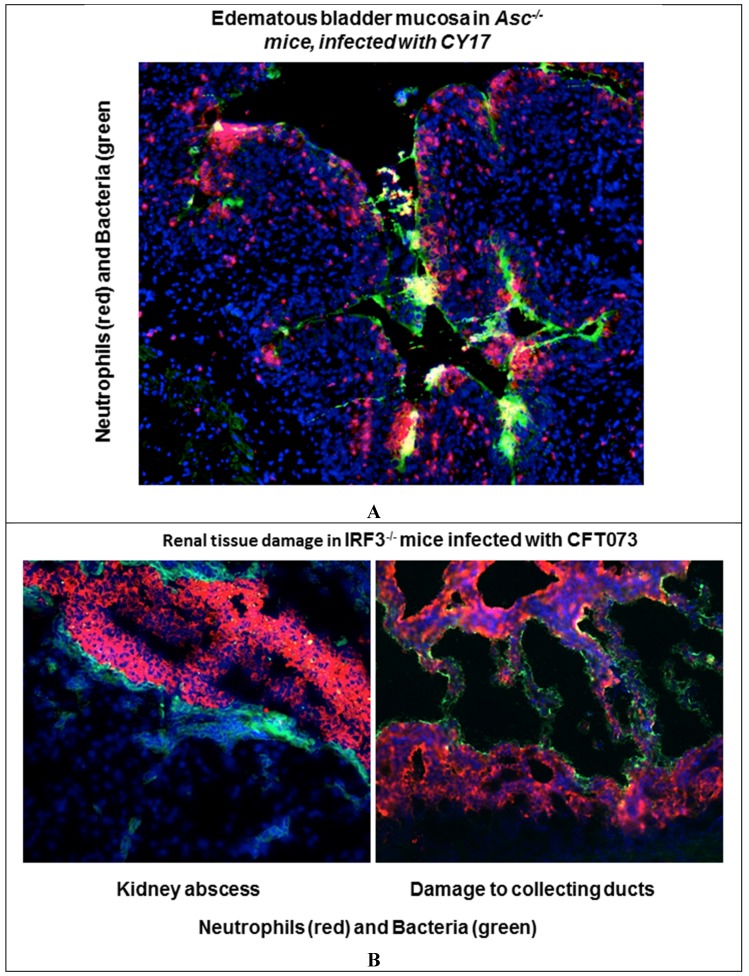
Inflammatory infiltrates in bladder and kidney tissue sections from infected mice with genetic deficiencies that enhance susceptibility to infection. Staining with specific antibodies to neutrophils (red) or *E. coli* (green). Nuclei are counterstained with DAPI. (**A**) Edematous bladder mucosa in *Asc^-/-^* mice infected with the acute cystitis strain *E. coli* CY-17; (**B**) Renal tissue destruction in *Irf3^-/-^* mice^,^ after infection with the acute pyelonephritis strain *E. coli* CFR073.

Inflammation and disease is essentially a product of a failed innate immune response (Figure 1). The importance of innate immunity for host resistance against UTI was first discovered in the early 1980s in the murine UTI model [21]. We found that C3H/HeJ mice had increased UTI susceptibility, defined by delayed bacterial clearance and impaired inflammation. As a result, C3H/HeJ mice remained chronically infected, without evidence of tissue damage. The “Lps” gene defect in C3H/HeJ mice was subsequently identified by Beutler *et al.* [22] and the importance of Tlr4 for host resistance to infection has since been confirmed in numerous models [19,23,24,25,26,27,28].

The observation in C3H/HeJ mice was conceptually important, as it demonstrated that the susceptibility to UTI is controlled by the host inflammatory response—later named “innate immunity” [23]. It also showed that UTI susceptibility is determined by the function of specific genes that control this innate immune response. A number of genetic defects have since been shown to influence UTI susceptibility in animal models and clinical studies (reviewed in [29]). For example, mCxcr2−/− mice with a neutrophil migration- and activation deficiency, develop severe APN with urosepsis followed by abscess formation and renal scaring [30,31,32]. Irf3^−/−^ mice develop a similar phenotype with over-activation of the innate immune response, severe, acute disease with urosepsis and abscess formation within one week of infection [19]. Mutant mice lacking Tlr5, Tlr11, Thp and cytochrome c oxidase subunit II (Cox2) are also more susceptible to UTI than mice with the wild-type genotype.

The strong effects of single genes were unexpected and controversial, as UTI susceptibility was thought at the time to be too complex to be controlled by single genes (reviewed in [29]).

Many of the identified genes influence the function of neutrophils, which are crucial effectors of the antibacterial defense in the urinary tract. Neutrophils are rapidly recruited by the chemotactic gradient emerging from the infected urothelium. Infection activates a broad chemokine response to infection, including CXC (CXCL1, CXCL5, CXCL8, CXCL9, and CXCL10) and CC chemokines (CCL2, CCL3, and CCL5) [33,34]. The main neutrophil chemoattractant is CXCL8 chemokine family, which guides neutrophil migration into the mucosa and the exit across the mucosa into the urine, which is a prerequisite to maintain tissue homeostasis [35,36]. Furthermore, activated neutrophils provide signals for the activation and maturation of macrophages and other mucosal cells, such as mast cells (see Abraham *et al.*, this Special Issue [1]). TNF-mediated crosstalk between neutrophils and macrophages has been shown to optimize the antibacterial defense in the urinary tract (see Zec *et al*., this Special Issue [1]).

## 5. Relevance of Genetic Variants for Disease in Susceptible or Resistant Patient Groups

Genes that control UTI susceptibility in mice are also polymorphic in UTI prone patients [29]. Inheritance of UTI susceptibility was confirmed in a three-generation family study, with low CXCR1 expression in the susceptible cases in those families. In contrast to classical human immunodeficiencies, the identified UTI susceptibility determinants do not primarily affect structural genes, but alter regulators of transcriptional efficiency.

For example, ABU patients carry TLR4 promoter genotype variants that lower TLR4 expression [37]. Intronic and 3′UTR variants in patients with acute pyelonephritis reduce CXCR1 transcription efficiency or RNA stability, permitting the infected host to regulate responses to a family of CXC chemokines that converge on this receptor [38]. In addition, we have identified promoter sequence variants that reduce the expression of Irf3, a key transcription factor that controls the TLR4-dependent response to uropathogenic bacteria. Transcriptional regulation provides a mechanism of genetic regulation different from the rare monogenetic disorders that affect innate immunity. In addition to the rare, complete loss of structural gene function, evolution appears to favor changes in the genetic make up, with less dramatic consequences for infected hosts [20,37,38].

## 6. Address how ABU Strains Achieve the State of “Commensalism”

For articles addressing this point, please see Dobrindt *et al.*, Ambite *et al*. and Wullt *et al.* in this Special Issue [1].

## 7. Immunomodulatory Therapies as a Complement to Antibiotics

As illustrated above, innate immunity provides an immediate defense against UTI. In resistant individuals, specific recognition and activation strategies eliminate pathogens while sustaining symbiosis with commensals. As the innate immune response relies on inflammatory pathways to execute defense functions, the activation of innate immune defenses has negative consequences, including destruction of host tissues. This lack of specificity and the genetic defects that upset the response, contribute to the pathobiology of disease.

The two-faced nature of innate immunity complicates the development of immune-modulatory therapies. An important challenge is to identify molecular targets that can be exploited to attenuate destructive inflammatory responses while accentuating those that are protective. The intervention needs to be precise, so that symptoms and tissue destruction caused by exaggerated host responses can be isolated and avoided.

We have identified two novel targets for immuno-modulatory therapies in UTI:

1. siRNA interference to attenuate the exaggerated inflammatory response in Irf3−/− mice with severe acute pyelonephritis.
2. Inhibitors of IL-1 and IL-1 processors, to attenuate the IL-1-dependent hyper-inflammatory state in acute cystitis.

Therapeutic efficacy has been demonstrated in the murine UTI model with promising results for translation into the clinic. To clarify, the term immunomodulation means that the immune system is specifically modified by interference with molecular regulators of the immune response, not by molecules with a more general mechanism of action, such as hormones, vitamins or food stuffs that cause numerous and complex changes in the host. 

In addition, preventive approaches are being explored, such as the development of vaccines against iron-binding proteins (Mobley *et al.* this Special Issue) or small molecule inhibitors of type 1 fimbrial binding to host cells (Hultgren *et al.*, this Special Issue).

## 8. Conclusions

Despite their prevalence and serious consequences for patients and society, UTIs are often not handled optimally. Ironically, the pace of change may be increased by the rapid emergence of antibiotic resistance in uropathogenic bacteria. The patients need alternative therapeutic approaches, in order to avoid a return to UTI-associated mortality, end stage renal disease and abortions or premature delivery. The increasingly problematic question is—how do we treat UTI and who do we treat with what?

Provided that the molecular findings discussed in this Special Issue are used, it will be possible to diagnose UTI susceptibility with better accuracy and to devise new therapeutic tools (Connolly *et al.* and Chao *et al.* in this Special Issue). For example, important diagnostic questions can be addressed with better accuracy. Bacterial virulence typing may be used to determine if fever in a patient with bacteriuria is evidence of acute pyelonephritis or ABU with fever of other origin. Infants and children with the first episode of acute pyelonephritis can potentially be identified, using genetic and proteomic tools. Genetic and proteomic screens may also be used to reduce the need for invasive imaging procedures and to increase predictive accuracy. Another relevant clinical question is the risk for postoperative infections in patients undergoing urogenital surgery.

Immunomodulatory therapy has great potential, especially if it will be possible to identify therapeutic targets that selectively attenuate “bad inflammation” and control destructive innate immune responses in kidneys and bladders. The development of specific inhibitors of “bad inflammation” will be essential, as well as clinical studies, to validate their function in relevant animal models.

## Figures and Tables

**Table 1:** The individual papers in this Special Issue of Pathogens, present in greater detail the results and the resulting advances in understanding of the molecular basis of UTI.

Name	Title
Bruce Beutler	KEY NOTE LECTURE Pathogens, Commensals, And Immunity: From the Perspective of The Urinary Bladder
	HOST SUSCEPTIBILITY TO INFECTION
Catharina Svanborg	Urinary Tract Infection Molecular Mechanisms and Clinical Translation
David Hains	Genetic Variation in Vesicoureteral Reflux and its Sequelae
Christian Kurts, Daniel Engel	Neutrophil-Migration into the Infected Uroepithelium is Regulated by the Crosstalk between Resident and Helper Macrophages
	HOST RESPONSE MODULATION BY BACTERIA
Soman Abraham	Why Serological Responses During Cystitis are Limited
Thomas Miethke	A comparative analysis of the mechanism of Toll-like receptor-disruption by TIR-containing protein C from uropathogenic *Escherichia coli* *
Ines Ambite	Bacterial control of host gene expression through RNA polymerase II *
David Hunstad	Subversion of host innate immunity by uropathogenic *Escherichia coli* binocular
	ASYMPTOMATIC BACTERIAL CARRIAGE
Lindsay Nicolle	The Paradigm Shift to Nontreatment of Asymptomatic Bacteriuria *
Björn Wullt	Asymtomatic Bacteriuria as a Model to Study the Coevolution of Hosts and Bacteria
Tommaso Cai	Asymptomatic bacteriuria in clinical urological practice: antibiotic preoperative prophylaxis and treatment of recurrent UTI
	BACTERIAL VIRULENCE
Harry Mobley	Measuring E. coli Gene Expression During Human Urinary Tract Infections
Matthew Mulvey	Histone Deacetylase 6 Regulates Bladder Architecture and Host Susceptibility to Uropathogenic *Escherichia coli*
Swaine Chen	Application and optimization of relE as a negative selection marker for making definitive genetic constructs in uropathogenic *Escherichia coli* strain UTI89
Swaine Chen	Brighter fluorescent derivatives of UTI89 utilizing a monomeric vGFP
Eric Klein	Perspective: Adhesion mediated signal transduction in uropathogenic *E. coli*
	NOVEL THERAPEUTIC APPROACHES
Annelie Brauner	Novel Strategies in the Prevention and Treatment of Urinary Tract Infections
Ann Stapleton	Cytoprotective effect of Lactobacillus crispatus CTV-05 against uropathogenic *Escherichia coli* *
Scott Hultgren	Adhesive Pili in UTI Pathogenesis and Drug Development *
Susanne Geerlings	Non-antibiotic prophylaxis for urinary tract infections *
Harry Mobley	Development of a Vaccine against *Escherichia coli* Urinary Tract Infections
Clara Maria Pichl	Biomickry of UPEC cytoinvasion: a novel concept for improved drug delivery in UTI
	ANTIBIOTIC RESISTANCE
Mark Schembri	Molecular characterization of the multidrug resistant *E. coli* ST131 clone
Florian Wagenlehner	The Global Prevalence of Infections in Urology (GPIU) study: A long term, world wide surveillance study on urological infections

The papers are available on the Special Issue website: http://www.mdpi.com/journal/pathogens/special_issues/urinary-tract-infection [1]. *: the papers are in press and would be online soon.
